# Editorial: Etiological mechanisms and treatments of idiopathic sudden sensorineural hearing loss

**DOI:** 10.3389/fneur.2023.1292836

**Published:** 2023-09-28

**Authors:** Xuewen Wu, Agnieszka J. Szczepek, Hajime Sano, Yong Feng

**Affiliations:** ^1^Department of Otolaryngology Head and Neck Surgery, Xiangya Hospital, Central South University, Changsha, Hunan, China; ^2^Department of Otorhinolaryngology, Head and Neck Surgery, Charité-Universitätsmedizin Berlin, Corporate Member of Freie Universität Berlin, Humboldt-Universität zu Berlin, Berlin, Germany; ^3^Department of Rehabilitation, Kitasato University Faculty of Allied Health Sciences, Kanagawa, Japan; ^4^Department of Otorhinolaryngology, University of South China Affiliated Changsha Central Hospital, Changsha, Hunan, China

**Keywords:** idiopathic sudden sensorineural hearing loss, hyperbaric oxygen therapy, steroids, magnetic resonance imaging, batroxobin

As a common otological emergency, idiopathic sudden sensorineural hearing loss (ISSNHL) occurs suddenly and without any prior warning, and is defined as hearing loss of at least 30 dB in three sequential frequencies within 72 h. The incidence of ISSNHL in Western countries was estimated to be 5–20 per 100,000 population (1). More recent investigations showed the annual incidence of ISSNHL to be 60.9 per 100,000 in Japan and 2.4–19 per 100,000 in some provinces of China, respectively (2–4). ISSNHL can be caused by a variety of factors, including vascular disorders, viral infections, and autoimmune diseases. However, the etiology and exact pathophysiology remain unclear in most cases, making the development of effective treatments challenging. This Research Topic aimed to gather recent findings that elaborate on the advanced etiological mechanisms and treatments of ISSNHL.

According to clinical practice, 3D-FLAIR magnetic resonance imaging (MRI) evaluation is an option for evaluating inner ear disturbances. It was reported that high signals can be found in the inner ear on 3D-FLAIR MRI in ISSNHL patients (5). In this Research Topic, Sone et al. review related clinical articles focusing on high signal and endolymphatic hydrops (EH) in the inner ear and describe the proposed pathophysiology of ISSNHL using cutting-edge 3D-FLAIR MRI evaluation. They find that different high signals on 3D-FLAIR MRI at different times represent different pathological patterns of the inner ear. For example, a pre-contrast high signal may indicate minor hemorrhage or increased permeability of surrounding vessels to the perilymph, whereas a post-contrast high signal indicates a breakdown of the blood–labyrinth barrier. In addition, primary EH could be pre-existing in some cases and may be a risk factor for the onset of ISSNHL. The authors suggest that the cutting-edge 3D-FLAIR MRI could be adopted as an evaluation method for elucidating the pathophysiology and predicting prognosis in ISSNHL.

Although the most frequently used treatment for ISSNHL is the administration of steroids (6, 7), other various empirical treatments also have been applied by some doctors in many countries, including hyperbaric oxygen therapy (HBOT), vasodilators, defibrinogen, and anticoagulant agents. Over the last two decades, HBOT and intratympanic steroids (ITS) were proposed as salvage treatments in cases of failure of systemic steroids (8, 9). Although HBOT and ITS have been proposed as optional treatments for ISSNHL patients in Europe and the United States more recently (10–13), there is still no broad, unanimous consensus about the efficacy of these treatments. In this Research Topic, Skarzynski et al. perform a retrospective study to comparatively investigate the efficacy of HBOT as an adjunct to glucocorticoid treatment in 63 adult ISSNHL patients. In this study, they do not find a beneficial effect of HBOT for ISSNHL. Mariani et al. perform a retrospective study on 75 ISSNHL patients to investigate the efficacy of HBOT and ITS in addition to systemic steroids. They find that the salvage treatments, both HBOT and ITS associated with systemic steroids, have similar hearing outcomes with no statistical differences as consecutive systemic steroids. These two articles indicate that HBOT or ITS may be of little benefit as salvage treatments.

Defibrinogen therapy for ISSNHL is also controversial. Some studies have indicated that intravenous batroxobin, a kind of defibrinogen medicine, may be effective in treating patients with ISSNHL (14, 15). The reduction of fibrinogen was presented as one of the main treatments for ISSNHL in the German Guideline (16, 17). Intravenous batroxobin was also recommended in the treatment of flat-type and total-deafness ISSNHL in the Chinese Guideline (18, 19). However, defibrinogen therapy was strongly recommended against for ISSNHL patients in the American Practice Guideline (1). In this Research Topic, Jiang et al. conduct a retrospective propensity score-matched study on 162 ISSNHL patients to investigate whether treatment combined with intravenous batroxobin is better than treatment without batroxobin. They find that there is no significant difference in short-term hearing outcomes between treatment with batroxobin and treatment without batroxobin in ISSNHL patients. Thus, the efficacy of batroxobin for the treatment of ISSNHL still remains to be determined.

In general, the etiology and exact pathophysiology of ISSNHL remain unclear, and the development of standardized treatments is also hampered. The efficacy of HBOT and ITS as salvage treatments and the long-term efficacy of batroxobin still need to be confirmed with larger randomized controlled trials. As research continues to uncover the underlying causes of ISSNHL, it may be possible to develop more targeted and effective treatments for ISSNHL.

## Author contributions

XW: Writing—original draft, Writing—review and editing. AS: Writing—review and editing. HS: Writing—review and editing. YF: Writing—review and editing.
